# Commentary: Antibodies to Human Herpesviruses in Myalgic Encephalomyelitis/Chronic Fatigue Syndrome Patients

**DOI:** 10.3389/fimmu.2020.01400

**Published:** 2020-07-23

**Authors:** Maria Eugenia Ariza

**Affiliations:** ^1^Department of Cancer Biology and Genetics, Wexner Medical Center, The Ohio State University, Columbus, OH, United States; ^2^Institute for Behavioral Medicine Research, Wexner Medical Center, The Ohio State University, Columbus, OH, United States

**Keywords:** Serology, EBV - Epstein-Barr Virus, Human herpesvirus (HHV)-6, Varicela zoster virus (VZV), Herpes Simplex virus type 1 and type 2 (HSV-1/2), Cytomegalovirus (CMV), Human herpesvirus 7 (HHV-7), Myalgic encephalomyelitis/chronic fatigue syndrome (ME/CFS)

## Introduction

Studies to ascertain a possible relationship between herpesviruses and myalgic encephalomyelitis/chronic fatigue syndrome (ME/CFS) have relied heavily on classical approaches, specifically, serological examination for antibodies against virus proteins, primarily structural, and/or increases in viral load (1–21). These data have been conflicting due in part to several features: the heterogeneity of the disease, high prevalence of the herpesviruses in the population since they can establish lifelong infections, and differences between laboratories. Two additional problems lead to conflicting data in serological studies: which viral antigens are to be used for detection, and what is the possible relationship, if any, of these viral antigens to ME/CFS? These are important questions that must be addressed for any data to provide meaningful insight into the possible contribution of a virus to the pathophysiology of ME/CFS. Although the experimental techniques used in Blomberg's serological study were appropriate, the selection of specific herpesviruses and viral antigens studied gives a limited view of the humoral response in ME/CFS.

## Discussion

**Figure 1:**
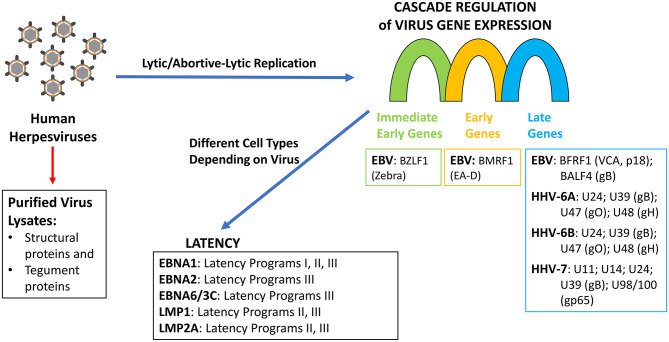
Herpesviruses replication and latency programs indicating the gene/gene products tested as antigens by Blomberg et al. (22).

Blomberg et al. (22) used a suspension multiplex immunoassay to detect antibodies against various herpesviruses' antigens, derived from proteins expressed during latency or late lytic replication (Figure 1), with the goal of determining differences in antibody titers against these antigens between ME/CFS patients and controls. However, no rationale was given as to why these particular antigens were chosen and what association, if any, they may have with ME/CFS. This is important because the antigenic properties of the different virus proteins are not the same. As demonstrated in an eloquent study by Vaider-Shalt et al. (23), over the course of their evolution, herpes simplex virus 1 (HSV-1), Epstein-Barr virus (EBV), human herpesvirus 8 (HHV-8), and human cytomegalovirus have decreased the number of epitopes present in virus proteins in order to help them avoid immune detection. Thus, the ability of a virus protein to generate an antibody response is dependent upon the amount of protein present in the host and its antigenicity. It is also not clear why Blomberg et al. (22) included HSV-1/2, human cytomegalovirus, HHV-7, and varicella zoster virus (VZV) in their study since there are no up-to-date literature reports establishing a serological relationship between these viruses and ME/CFS.

Along these lines, measuring the response in patients' sera using purified virus lysate would only detect antibodies to proteins that are components of the virion (capsid proteins, glycoproteins, and tegument proteins) but not proteins expressed during virus lytic/abortive-lytic replication (Figure 1). Furthermore, since herpesviruses establish persistent infections in a significant proportion of the population (24), it is not surprising that antibody titers against purified virus lysates are not significantly different between ME/CFS cases and controls, especially since viremia has not been demonstrated in ME/CFS patients. Using “crude” antigen preparations make it impossible to ascertain whether there is a difference in the formation of specific antibodies against a single protein within the two populations under study. Although the epitope mapping studies provide qualitative data, the antigens have little relevance to ME/CFS, as they focused primarily on viral proteins that are expressed either during latency in the case of EBV or late in the lytic replicative cycles of EBV, HHV-6A/B, and HHV-7. Several studies have demonstrated that there is no increase in viral load of EBV or HHV-6A/B in ME/CFS patients (12, 14, 15, 17, 21), which suggests that there is no increase in the expression of late herpesvirus proteins in ME/CFS patients. Nor is there, in the case of EBV, an increase in viral capsid antigen (VCA) antibody titers, suggesting that abortive lytic replication is occurring in these patients (1, 17–19). Likewise, there is no evidence suggesting a role of any latency expressed gene products in ME/CFS. There is a single report showing a small non-significant difference in IgG response to Epstein-Barr nuclear antigen 3C (EBNA-3C; EBNA-6) by peptide microarray (25). EBNA-3C is the product of a gene only expressed in B cells undergoing the latency III gene-expression program, and there is no evidence to support latency type III in ME/CFS. Similarly, no rationale was provided for examining EBNA-1, although there are no studies linking it with ME/CFS. If a causal relationship is to be established between EBV, HHV-6, or any other herpesvirus and ME/CFS, it will require addressing the role of viral proteins produced during abortive-lytic replication of the herpesviruses, which does not lead to new viral progeny, and thus, no changes in viral load would be observed. Therefore, measuring humoral responses in patients' sera using virus lysate is not appropriate because it would only detect antibodies to proteins that are components of the virion but not to proteins expressed during abortive-lytic replication.

Lastly, Blomberg et al. (22) indicate in the *Introduction* and *Discussion* of their manuscript that a recent study (1) implicated VZV in ME/CFS. That is not the case. In that study, the investigators demonstrated that although ME/CFS patients' serum contained anti-VZV deoxyuridine triphosphate nucleotidohydrolase (dUTPase) antibodies, it was not significant (*p* < 0.857).

In conclusion, although the suspension multiplex immunoassay seems appropriate, it may lack the sensitivity of the luciferase immunoprecipitation system (26) and VirScan (27) serological platforms that have been utilized for the detection of viral infections including those caused by EBV, HSV-1, HSV-2, HHV-8, and VZV (28).

## Author Contributions

The author confirms being the sole contributor of this work and has approved it for publication.

## Conflict of Interest

The author declares that the research was conducted in the absence of any commercial or financial relationships that could be construed as a potential conflict of interest.
